# Tree species richness predicted using a spatial environmental model including forest area and frost frequency, eastern USA

**DOI:** 10.1371/journal.pone.0203881

**Published:** 2018-09-18

**Authors:** Youngsang Kwon, Chris P. S. Larsen, Monghyeon Lee

**Affiliations:** 1 Department of Earth Sciences, University of Memphis, Memphis, Tennessee, United States of America; 2 Department of Geography, University at Buffalo, Buffalo, New York, United States of America; 3 Geospatial Information Sciences, University of Texas at Dallas, Richardson, Texas, United States of America; Universidade Federal de Uberlandia, BRAZIL

## Abstract

Assessing geographic patterns of species richness is essential to develop biological conservation as well as to understand the processes that shape these patterns. We aim to improve geographic prediction of tree species richness (TSR) across eastern USA by using: 1) gridded point-sample data rather than spatially generalized range maps for the TSR outcome variable, 2) new predictor variables (forest area FA; mean frost day frequency MFDF) and 3) regression models that account for spatial autocorrelation. TSR was estimated in 50 km by 50 km grids using Forest Inventory and Analysis (FIA) point-sample data. Eighteen environmental predictor variables were employed, with the most effective set selected by a LASSO that reduced multicollinearity. Those predictors were then employed in Generalized linear models (GLMs), and in Eigenvector spatial filtering (ESF) models that accounted for spatial autocorrelation. Models were evaluated by model fit statistics, spatial patterns of TSR predictions, and spatial autocorrelation. Our results showed gridded TSR was best-predicted by the ESF model that used, in descending order of influence: precipitation seasonality, mean precipitation in the driest quarter, FA, and MFDF. ESF models, by accounting for spatial autocorrelation, outperformed GLMs regardless of the predictors employed, as indicated by percent deviance explained and spatial autocorrelation of residuals. Small regions with low TSR, such as the Midwest prairie peninsula, were successfully predicted by ESF models, but not by GLMs or other studies. Gridded TSR in Florida was only correctly predicted by the ESF model with FA and MFDF, and was over-predicted by all other models.

## Introduction

Spatial variation in species richness has been studied for over 200 years [1]. Causes of these variations have been explored for many taxonomic groups, from microscopic to global scales, using descriptions, experiments, and spatial environmental models (hereafter “models”). Many factors have been forwarded to explain spatial variation in species richness: environment, ecology, area, and evolution [2–4]. However, improved ability to predict species richness is required to better-model its spatially heterogeneous responses to global change [5]. In this study we model spatial variations in gridded tree species richness (TSR) at the subcontinental scale.

Eight previous studies that modeled TSR found different single-best predictors: actual evapotranspiration [6,7], net primary productivity [8], median temperature [9], glacial history [10], accessibility [11], mean temperature of coldest quarter of the year [12], and phylogenetic species variability [13]. Multivariate models have explained much variance in TSR: 74.0% [10], 81.5% [11], 83.2% [12], 84.7% [7], 86% [6], 97.1% [13], and 97.5% [8]. Those studies employed 50 different predictor variables, from four [7,11] to 25 [12], with few variables common to multiple studies.

Despite the high predictive power of those models, geographic errors in TSR predictions have been little considered. Only Wang et al. [12] considered them, though they only noted their over-prediction of TSR in Florida. Their model also failed to predict areas of low TSR in the Midwestern prairie peninsula [14], along the lower Mississippi River, and along the Gulf Coast, that were apparent in their map, and that of Montoya et al. [10], of TSR input data created using species range maps [15]. We aim to improve subcontinental predictions of TSR in four regards: using point-sample data rather than range maps for the TSR outcome variable, using additional predictor variables, addressing spatial autocorrelation, and considering geographic error.

First, for the TSR outcome variable, all eight aforementioned TSR studies estimated it by overlaying spatially generalized species range maps, such as those of Little’s range maps [15]. However, range maps are well-known to overestimate area of a species’ occupancy for trees [16] and animals [17]. For example, Little’s range maps are produced as spatially generalized polygons delineated from published tree distributions, herbarium records, and field surveys of varying sample intensity. Further, range maps exhibit higher spatial autocorrelation than do point-sample data, resulting in artificially inflated variance explained by spatial environmental data [18]. Use of TSR point-sample data should thus improve spatial accuracy of TSR predictions.

Second, although subcontinental predictions of TSR in the eight aforementioned studies already employed 50 predictor variables, models might be improved by inclusion of two additional variables: forest area (FA) and frost frequency. In regards to FA, when TSR data were gathered from irregular-sized study areas, TSR was related to study area size [19,20] as expected from species-area relations [21]. The collection of TSR data into regular-sized grids, such as employed in the eight aforementioned TSR studies, might be thought to remove the species-area relationship. However, since grids contain variable amounts of FA, due to presence of waterbodies and crops, a species-FA relation might remain in gridded TSR data. In regards to frost, Fine [3] outlined how frost may influence TSR as tropical tree species are susceptible to frost, and as temperate tree species incur reduced growth rates to increase frost resistance. Relatedly, Wang et al. [12] found that TSR was strongly and positively related to mean temperature in the coldest quarter, and suggested that it was due to colder winters having more frost days. If that is the case, we suggest it would be more appropriate to employ a quantitative measure of frost incidence because, for example, continental areas should have more frost days than maritime areas with the same mean winter temperature [22].

Third, if spatial autocorrelation is present in the predictor or outcome variables, it may violate the statistical assumption that observations are independent and identically distributed, and may thus lead to biased parameter estimates [23]. Since most species and environmental data exhibit spatial autocorrelation, and since the species range maps employed by all previous TSR studies have additional spatial autocorrelation [18], the modeled predictions of TSR might contain much error. Spatial autocorrelation has been accounted for in models of species richness of vascular plants [24], reptiles [25] and various vertebrate groups [4], but in models of TSR only by Svenning & Skov’s work [11]. Models that account for spatial autocorrelation explain more variance and have lower spatial autocorrelation than do general linear models [24].

We will model TSR across eastern USA and evaluate it for geographic errors. We hypothesize that geographic predictions of TSR for eastern USA will improve through the use of gridded, FIA point-sample data as the outcome variable, forest area and frost frequency included as predictor variables, and a regression model that accounts for spatial autocorrelation.

## Materials and methods

### Forest inventory and analysis (FIA) database

The FIA program of the USDA Forest Service conducts nation-wide field-measured tree point-sample surveys using a systematic five-year inventory [26]. Each FIA plot inventories trees in four 7.2 meter fixed-radius subplots. Privacy issues require the latitude and longitude of plots be perturbed up to 0.8 km, and that some plots on private lands have locations swapped [27]. The influence of perturbing and swapping should be negligible in this study since FIA plots were aggregated into 50 km by 50 km grids. The FIA database (FIADB, version 5.1) was downloaded for the 31 easternmost USA states from the FIA DataMart (https://apps.fs.usda.gov/fia/datamart/) for the five-years 2009 to 2013.

### Tree species richness (TSR)

TSR was estimated from FIA data, with individual plots aggregated into 50 km by 50 km grids, matching those of Wang et al. (2011). We included all trees (live trees with stems ≥12.7 cm DBH) from the four sub-plots. If a grid was fully forested it would contain 100 plots at the current FIA sampling intensity, though five states (Delaware, Indiana, Michigan, Rhode Island, and Wisconsin) employ double-sampling intensity. To account for variance in numbers of plots per grid, we employed non-parametric bootstrapping to estimate each grid’s mean TSR. We randomly selected, without replacement, the same number of plots from each grid 1000 times, giving 1000 TSR estimates. We tested three numbers of plots drawn 1000 times: 31 plots from the 1234 grids with ≥31 plots, 41 plots from the 945 grids with ≥41plots, and 51 plots from the 596 grids with ≥51 plots. The 134 grids with ≤30 plots were not employed. Further analyses focused on the 1234 grids with ≥31 plots, which together contained 79,145 FIA plots with 2,745,363 trees from 254 species. Bootstrapped estimates of TSR using 41 and 51 plots drawn from, respectively, grids with ≥41 and ≥51 plots, were strongly related (R^2^>0.96) to their TSR estimated using 31 plots. Using more grids provided a greater range of environmental conditions for parameterization of environmental models, and a larger area over which to assess geographic errors.

### Environmental predictor variables

Eighteen environmental predictor variables (Table 1) were grouped into seven categories: climatic seasonality, energy availability, energy-water dynamic, habitat heterogeneity, water availability, areal factors, and limiting climatic factors. The first five categories contain variables previously used to explain species richness [9,12]. The areal and limiting climatic categories were added to potentially account for over-predicted TSR in Florida. The 18 variables and seven categories were chosen to represent the variety of predictors and the specific variables that had been most successful. Temporal factors (e.g. glacial history or phylogenetic information) were not included due to incomplete data availability.

**Table 1:** GLM-based relations between the 18 individual predictor variables and tree species richness.

Category	Variable	Standardized coefficient	Deviance (%) explained
Areal factors	FA	0.172	30.31
	WA	-0.104	13.28
Climatic seasonality	ART	-0.128	21.36
	PSN	-0.170	31.85
	TSN	-0.109	20.72
Energy availability	MAT	0.122	23.59
	MTWQ	0.124	23.89
	PET	0.119	23.41
Energy-water dynamic	PET–PET2 + MAP	0.104	18.72
Habitat heterogeneity	RA	0.019	0.60
	RMAP	-0.102	18.07
	RMAT	-0.121	20.07
Limiting climatic factors	MFDF	-0.173	33.32
	MPDQ	0.174	32.28
	MTCQ	0.117	23.83
Water availability	AET	0.140	28.16
	AI	0.000	0.02
	MAP	0.132	24.31

P-values are less than 0.01 for all variables.

Forest Area (FA, km^2^) and Waterbody Area (WA, km^2^) were calculated from 1 km by 1 km resolution MODIS land-cover data (MCD12Q1). FA in each grid was calculated by overlaying all MODIS forest-related classes. MODIS land cover was employed instead of FIA plot counts to ensure independence of the FA predictor and TSR outcome variables. WA was calculated as water-cover classified in MCD12Q1 in a 100 km radius from the center of each 50 km by 50 km grid.

Monthly temperature and precipitation data (http://www.worldclim.org) were obtained for 1950 to 2000 with a 30 arc-second resolution (ca. 1 km at the Equator). Climate variables were created as means and differences (Table 1): Annual Range of Temperature (ART, ^o^C), Mean Annual Temperature (MAT, ^o^C), Mean Temperature of Warmest Quarter (MTWQ, ^o^C), Range of Mean Annual Precipitation (RMAP, mm), Range of Mean Annual Temperature (RMAT, ^o^C), Mean Precipitation of Driest Quarter (MPDQ, mm), Mean Temperature of Coldest Quarter (MTCQ, ^o^C), and Mean Annual Precipitation (MAP, mm). Precipitation Seasonality (PSN, mm) was the coefficient of variation (standard deviation of monthly precipitation totals divided by mean monthly precipitation) and Temperature Seasonality (TSN, ^o^C) was standard deviation of mean monthly temperature. Quarters of the year were three-month groupings: DJF, MAM, JJA, and SON.

Three climate variables with 30 arc-second resolution were downloaded from CGIAR (http://www.cgiar-csi.org): Mean frost day frequency (MFDF, days per month) calculated as mean days per month for 1901 to 2006, and Potential evapotranspiration (PET, mm) and Aridity Index (AI, unitless) calculated as annual mean for 1950 to 2000. Actual evapotranspiration (AET, mm) with a 1 km by 1 km resolution was downloaded as annual mean from MODIS Global Evapotranspiration Project (http://www.ntsg.umt.edu) for 2000 to 2010. PET indicates net atmospheric energy balance independent of water availability, AET is amount of water actually removed, and AI indicates precipitation availability over atmospheric water demand [28]. A frost day occurred when minimum temperature was below 0°C, as modelled from monthly average daily minimum temperature [29].

Energy-water dynamics were evaluated using PET–PET^2^ + MAP (cf. Wang et al. [12]). Range of altitude (RA, m) was the difference between absolute minimum and maximum elevation in each 1 km by 1 km grid of a digital elevation model (https://lta.cr.usgs.gov/GTOPO30). Difference based variables (RA, RMAP, and RMAT) were aggregated into 50 km by 50 km grids as differences between absolute minimum and maximum values in all 1 km by 1 km grids within larger 50 km by 50 km grids. All other variables were aggregated into 50 km by 50 km grids as means of 1 km by 1 km climate or land-cover data within it following removal of outliers (including NA-coded values along coastline grids).

### Analytic methods

TSR predictions employed two steps. First, variables were selected using a Least Absolute Shrinkage and Selection Operator (LASSO; [30]). LASSO is superior to regular stepwise ordinary least squares in its handling of multicollinearity [31]. LASSO was used only for variable selection and not for TSR prediction because its model coefficients for selected variables are greatly penalized (described below), making its results difficult to compare with those from traditional regressions. Second, using the variables selected by LASSO, TSR was modelled using generalized linear models (GLM) and Eigenvector spatial filtering (ESF). These two steps are further detailed in the next three subsections.

### Variable selection

To reduce the number of predictor variables with multicollinearity, we used a LASSO under the GLM with logarithmic link function. LASSO uses a penalty term to constrain the size of estimated coefficients (i.e. *shrink* the effect of less important predictor variables) by minimizing the deviance (i.e. sum of squared differences) of the linear model fit to the observed outcome. LASSO penalizes the coefficients of unimportant variables to zero, resulting in a subset of predictor variables in the model. All continuous predictor variables were standardized (mean = 0, SD = 1) so regression coefficients could be compared as measures of relative importance [32]. The LASSO penalty parameter λ for variable selection was determined using a 10-fold cross-validation. We applied LASSO to two sets of variables: all predictors (“full model”), and all predictors except MFDF and FA (“reduced model”). LASSO was conducted using the R package *glmnet* [33].

### Generalized linear models (GLMs)

GLMs allow the distribution of the outcome variable to be modeled to fit any member of the exponential family [34]. We used a GLM with a Poisson error distribution and a logarithmic link function since TSR generally satisfies the Poisson error distribution as a count variable. We first ran GLMs for the 18 single predictor variables of TSR to evaluate their individual explanatory power. We then developed two sets of predictor variables using LASSO to obtain non-penalized coefficients and predictions: LASSO output from all 18 predictor variables (“full model”) and LASSO output from 16 predictor variables not including MFDF and FA (“reduced model”). Performance of these two models was assessed by the Akaike Information Criterion (AIC) value and residual deviance (% explained). GLM analyses were conducted using the R package *stats*.

### Eigenvector spatial filtering (ESF)

Two popular methods to account for spatial autocorrelation are simultaneous autoregressive models, and conditional autoregressive models. They both include a spatial autocorrelation parameter using a spatial weight matrix (usually a 0 and 1 binary contiguity matrix with a n-by-n dimension) on the right-hand side of the equation. These models can be specified in various ways based on which component has spatial autocorrelation [35]. Eigenvector spatial filtering (ESF; [36]) has become a more popular method as it successfully accounts for spatial autocorrelation in a regression model specification. Although the fundamentals of autoregressive-models and ESF are similar, ESF has advantages over autoregressive-models. In particular, ESF models are simpler as they capture spatial autocorrelation as a mean response rather than as variance [36]. Moreover, an autoregressive-Poisson model cannot account for negative spatial autocorrelation while ESF can through its integrability conditions [36]. Its flexible structure, which introduces eigenvectors extracted from a transformed spatial weights matrix using a stepwise selection procedure, facilitates different model specifications.

ESF is a nonparametric method that can be adapted to GLMs. It uses a set of eigenvectors extracted from the decomposition of (**I** − **11**^T^/n)**W**(**I** − **11**^T^/n), where **I** is an identity matrix, **1** is a vector of ones, n is the number of spatial units, **W** is the binary spatial weight matrix, and T is the matrix transpose operator. We extracted n eigenvectors from a n – by – n matrix, with all eigenvectors uncorrelated and orthogonal to each other. These eigenvectors can represent distinctive map patterns and be employed as components in a regression to capture spatial autocorrelation. The general ESF form is:

$$Y=X\beta_{x}+E_{k}\beta_{k}+\epsilon$$ (Eq 1)

where **β**_x_ is a set of parameter vector for predictor variables, **E**_k_ is a set of eigenvectors, and **β**_k_ is another set of parameter vector for **E**_k_. The set of eigenvectors are selected based on the significance level of the estimated coefficients using a stepwise regression procedure [37]. We utilize AIC for the model quality measure in each step of stepwise regression. ESF analyses were conducted using the R packages *MASS* and *spdep*.

### Model evaluation

The GLMs and ESF models, both full and reduced, were compared in several regards: variable multicollinearity as indicated by variance inflation factors (VIFs), model fit as indicated by AIC and percent of total deviance explained, and spatial autocorrelation of standardized residuals as indicated by Moran’s I. VIFs indicate if multicollinearity exists in a regression analysis by measuring how much the variance of estimated regression coefficients are inflated relative to when predictor variables are not linearly related. Total deviance explained by a model was calculated in two steps. First the difference between the deviance for the given model and the saturated model deviance was calculated as:

$$2\sum_{i=1}^{n}\left(y_{i}(\log\left(\frac{y_{i}}{u_{i}}\right)-(y_{i}-u_{i})\right)$$ (Eq 2)

Second, percent deviance explained was calculated: (100-null deviance/residual deviance)* 100.

Spatial autocorrelation was assessed in two steps. First, for each grid the standardized Pearson residual was calculated as its raw residual divided by the standard error of all residuals. Second, residuals for all 1234 grids were evaluated using Moran’s I.

TSR predictions made by our four models (GLM full, GLM reduced, ESF full, ESF reduced) and the model of Wang et al. (2011) were compared with gridded FIA TSR point-sample data using the non-parametric Wilcoxon signed-rank test, paired at the grid-scale. This was done as TSR data are not normally distributed. This test compared the predicted TSRs for each grid for two areas: Florida, and the inland eastern USA area not including Florida. The null hypothesis was that the median difference of TSR between predictions and FIA observations of matched grids was zero.

## Results

### Tree species richness (TSR)

**Fig 1:**
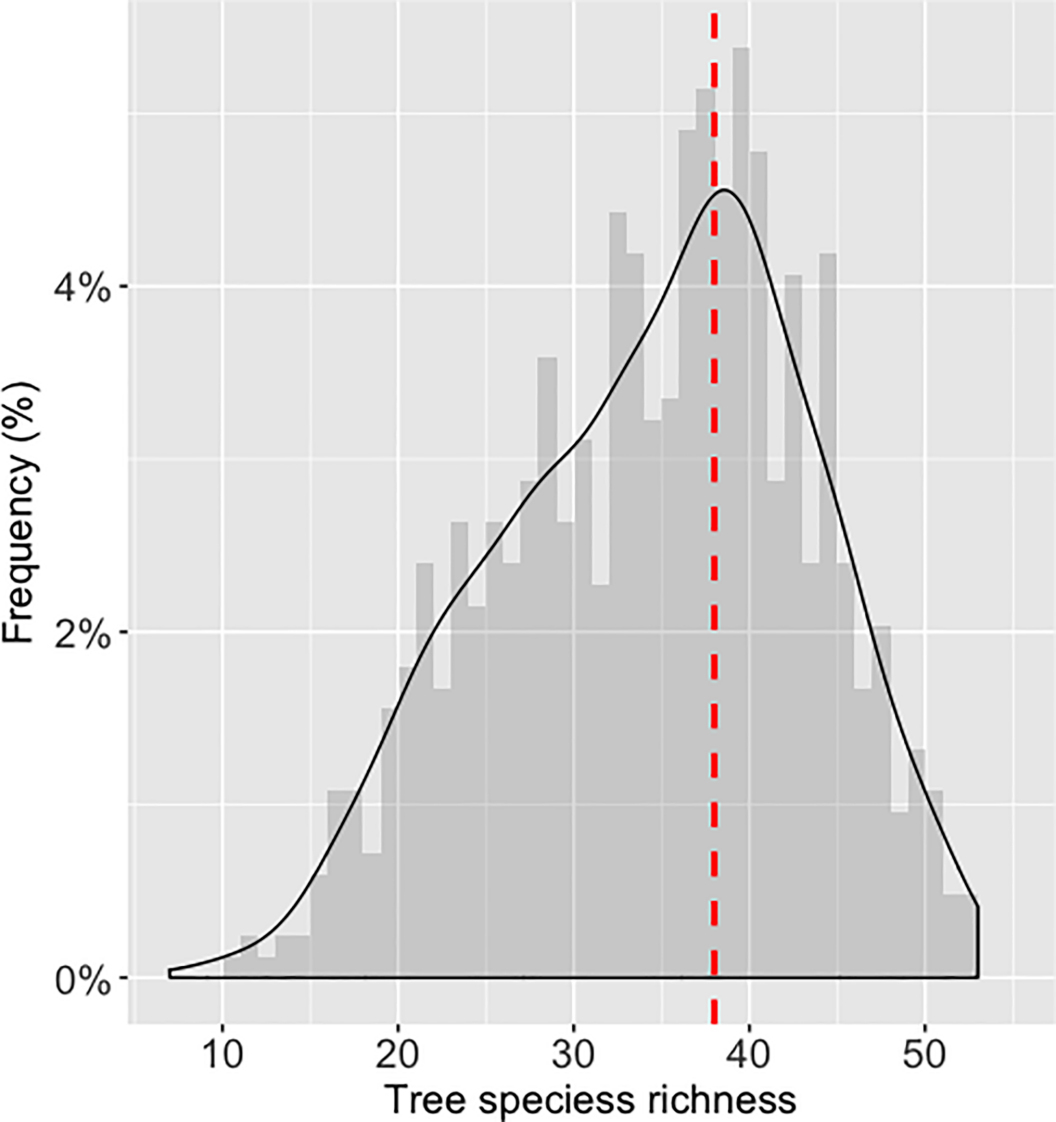
Frequency of tree species richness in the 1234 grids of 50km^2^. The smoothed line is the kernel density estimate; the red line indicates the median of 37.9 species.

**Fig 2:**
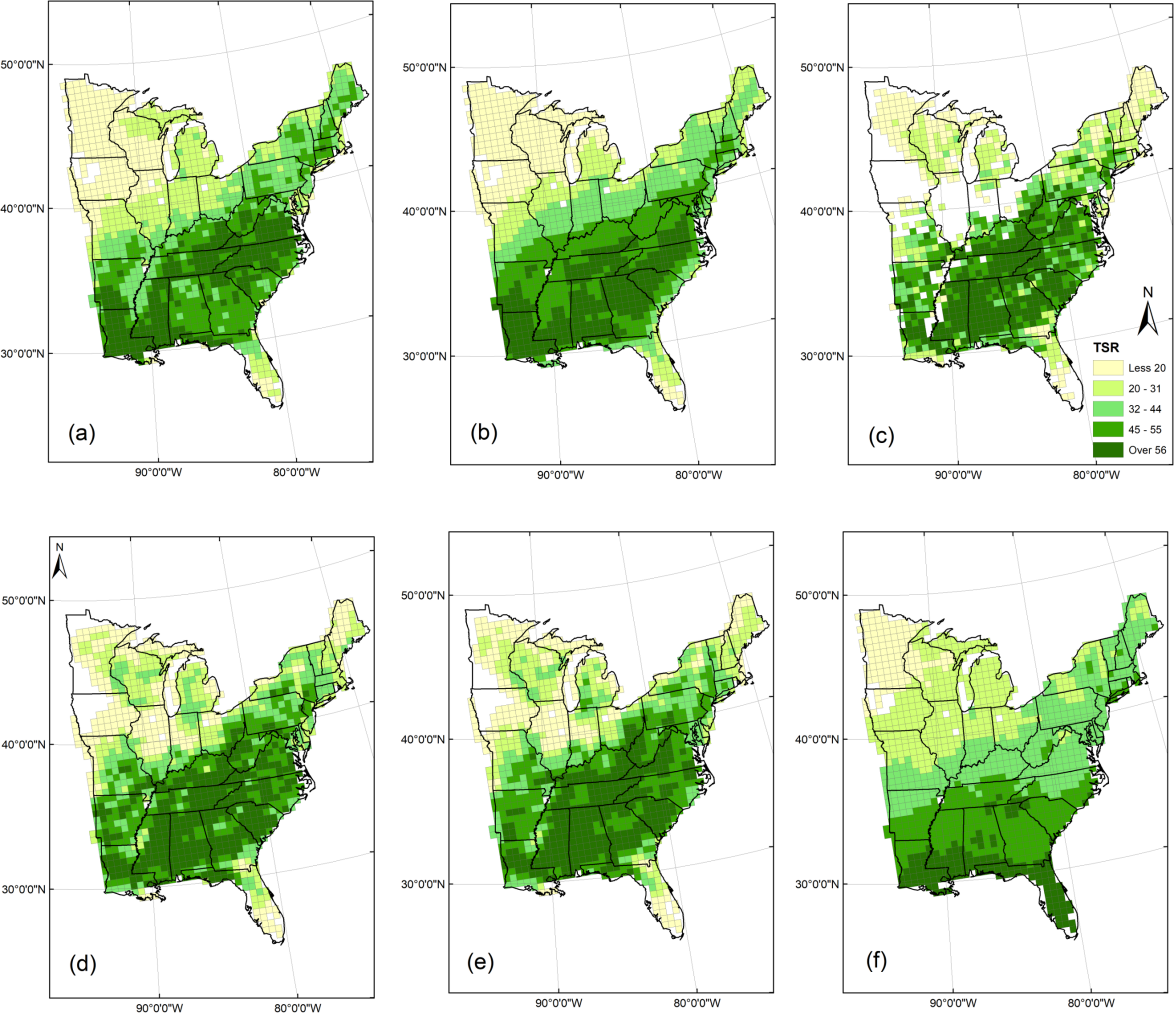
Tree species richness (TSR): observed by the FIA (c), and predicted by our GLMs, full (a), and reduced (b), our ESF models, full (d) and reduced (e), and by the model of Wang et al. (2011; f). The same TSR classes are employed in all maps.

TSR in the 1234 grids ranges between 6 and 52, has a mean of 36.4, a median of 37.9, and is left-skewed (Fig 1). TSR in gridded FIA point-sample data (Fig 2C) is low in the northern states and Florida, and high in the southern states though slightly lower along the Gulf Coast. Grids with no mapped TSR values contain <31 FIA plots and thus TSR could not be estimated; one block of them is along the lower Mississippi River, and a second block from Iowa to western Ohio corresponds with the Midwestern prairie peninsula [14].

### Variable selection

Assessed using individuals GLMs, five predictor variables each explained >25% of deviance in gridded TSR (presented in descending order): MFDF, MPDQ, PSN, FA, AET (Table 1). The absolute value of the standardized coefficient followed the same order as deviance explained. Relations with gridded TSR were negative for PSN and MFDF and positive for the other three predictors.

LASSO, using a 10-fold cross validation so that error was <1 S.E. of the minimum, selected four of the 18 variables for the full model and four of the 16 variables for the reduced model (Table 2). In the full model, the effect of variables, as indicated by the absolute size of standardized and penalized regression coefficients was, in descending order: MFDF, PSN, FA, MPDQ. For the reduced model, the effect of variables was, in descending order: PSN, PET, MPDQ, MTCQ. The variables common to the full and reduced models were PSN and MPDQ. All variables had the same sign in LASSO (Table 2) as in individual GLMs (Table 1) but, due to the LASSO penalization procedure, penalized standardized coefficients were smaller.

**Table 2:** Variables chosen by the LASSO method to reduce multicollinearity in the GLM.

Full model (variables selected from all 18 predictors)		Reduced model (variables selected from the 16 predictors other than MFDF and FA)	
Selected variables	Standardized penalized coefficient	Selected variables	Standardized penalized coefficient
MFDF	-0.0063613	PSN	-0.0056451
PSN	-0.0050020	PET	0.0031112
FA	0.0002133	MPDQ	0.0001843
MPDQ	0.0001359	MTCQ	0.0001213

Variables are ordered from highest to lowest absolute standardized coefficient.

### Model predictions

Gridded TSR was predicted best by the full ESF model as indicated by the highest percent deviance explained and lowest AIC value, second-best by the reduced ESF model, third-best by the full-GLM, and poorest by the reduced GLM (Table 3). Standardized regression coefficients indicate PSN was the most influential variable in all four models (Table 3), MFDF was second-most influential in the full GLM and fourth-most in the full ESF model, and FA was third-most influential in the full ESF model and fourth-most in the full GLM. For the reduced models, LASSO chose PET and MTCQ to replace FA and MFDF. The sign of the coefficients in all four models was the same as in individual GLMs (Table 1) and in LASSO variable choice (Table 2). VIF values for all four variables in all four models were less than five, indicating negligible multicollinearity among predictor variables [38].

**Table 3:** Model comparisons between GLM and ESF.

Full models (variables selected from all 18 predictors)						
Variables	GLM Poisson			ESF		
	Standardized Coefficient	Standard Error	VIF	Standardized Coefficient	Standard Error	VIF
(Intercept)	3.11841	0.003725***	NA	3.23212	0.0653319***	NA
PSN	-0.16512	0.006331***	3.66	-0.25411	0.0036453***	1.70
MFDF	-0.13262	0.003424**	1.41	-0.12807	0.0043615**	1.91
MPDQ	0.11226	0.017050 ***	4.11	0.16448	0.0197261***	4.48
FA	0.09734	0.004991***	2.36	0.15963	0.0151168**	3.71
AIC	9061			7715		
Null Deviance	6113			6113		
Residual Deviance (% explained)	2275 (62.78%)			749 (87.74%)		
Moran’s I	0.68			0.13		
Z-score of Moran’s I	23.893***			2.063*		
# selected eigenvectors	-			160		
Reduced models (variables selected from the 16 predictors other than MFDF and FA)						
Variables	GLM Poisson			ESF		
	Standardized Coefficient	Standard Error	VIF	Standardized Coefficient	Standard Error	VIF
(Intercept)	3.0027	0.00541***	NA	3.0095	0.01823***	NA
PSN	-0.1930	0.011649***	3.61	-0.19932	0.017165***	4.55
MPDQ	0.13483	0.017770**	4.14	0.07313	0.006333**	2.61
PET	0.10386	0.003566**	1.41	0.08173	0.003745**	1.80
MTCQ	0.08233	0.013201***	3.60	0.04512	0.004363***	1.92
AIC	9899			8019		
Null Deviance	6113			6113		
Residual Deviance (% explained)	3111 (49.10%)			1108 (81.87%)		
Moran’s I	0.75			-0.14		
Z-score of Moran’s I	28.90***			2.57**		
# selected eigenvectors	-			188		

Variables are ordered in descending order of absolute size of the standardized coefficient for the GLM; VIF is the variance inflation factor.

Significance level

***0.001

**0.01

*0.05

The spatial pattern of TSR predicted by all four models, similar to that in gridded FIA point-sample data, was low values in northern states, Florida, and along the Mississippi River, and high values in southern states (Fig 2). GLM and ESF predictions varied in four regards: ESF predicted lowered TSR in the prairie peninsula from Iowa to Ohio while GLMs did not, ESF predicted lower values of TSR along the Gulf coast while GLMs did not, ESF predicted minor reductions in TSR along the lower Mississippi River while GLMs predicted marked reductions, and ESF predicted spatially heterogeneous patterns of high TSR in southern states while GLMs predicted blocky patches of high TSR. In comparison, the model of Wang et al. [12] also did not predict lowered TSR in the prairie peninsula, along the lower Mississippi River, along the Gulf coast, or in Florida (Fig 2F).

**Fig 3:**
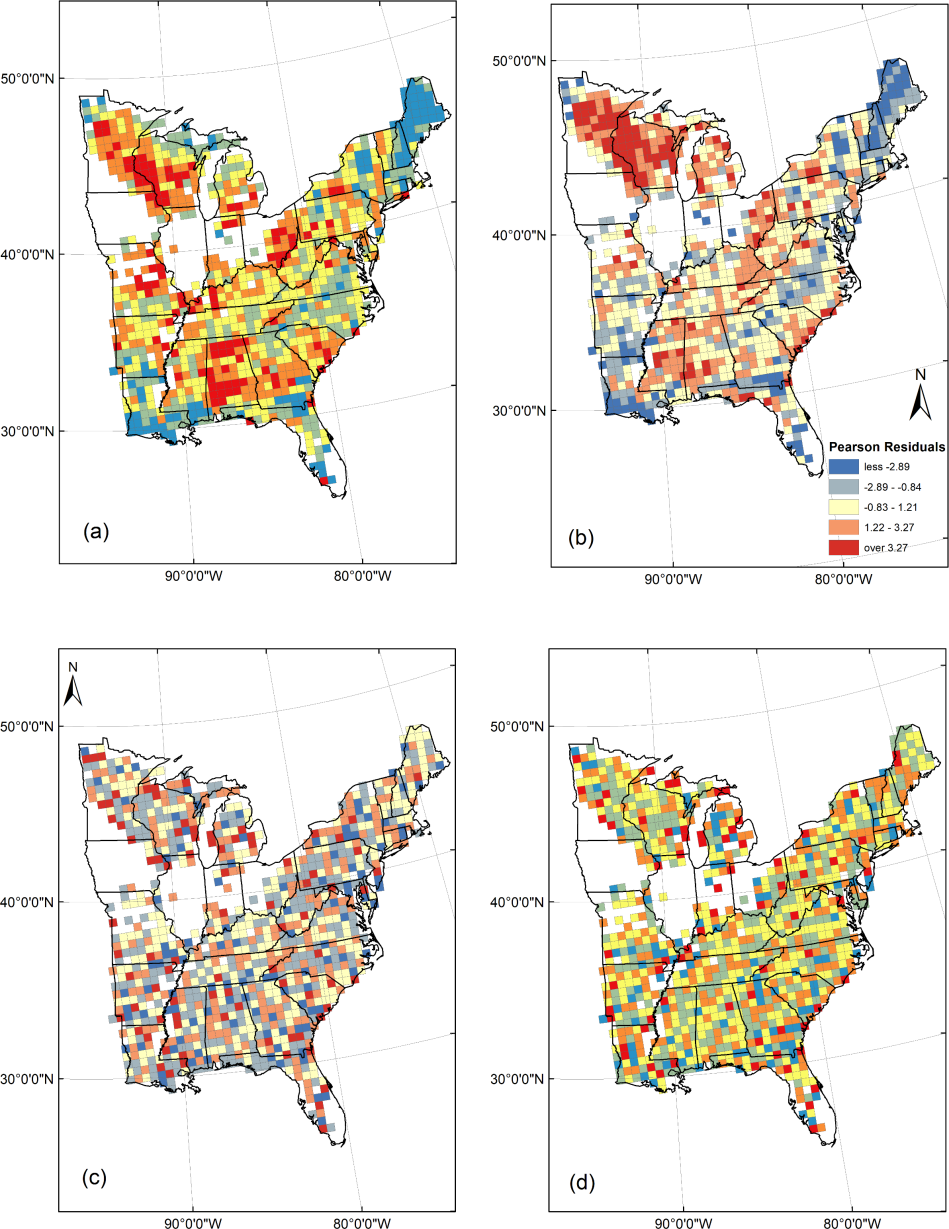
Standardized Pearson residuals for our two GLMs, full (a) and reduced (b), and two ESF models, full (c) and reduced (d). The same residual classes are employed in all maps.

The observed and z-scores of Moran’s I values indicate that standardized residuals were highly significantly clustered (positive) for both GLMs, but the majority of positive spatial autocorrelation was captured and slightly dispersed in both ESF models (Fig 3, Table 3). Predicted and FIA values of TSR at the grid-scale were not significantly different in Florida for the full ESF model, and not significantly different in inland Eastern USA for the full and reduced ESF models (Table 4). TSR was significantly over-predicted in Florida by the GLMs and the reduced ESF model and especially by Wang et al. [12]. TSR was significantly under-predicted in inland eastern USA by the two GLMs and especially by Wang et al. [12]. The inclusion of the two new predictors, FA and MFDF, improved prediction accuracy such that the full models of GLM and ESF had 13.7% and 5.9% higher deviance explained than their respective reduced models.

**Table 4:** Wilcoxon signed-rank test between FIA and predicted TSR for eastern USA and Florida.

Model	Eastern USA inland (Median = 39.2, N = 1182)			Florida (Median = 32.3, N = 52)		
	Median	W*	P-value (two-tailed)	Median	W*	P-value (two-tailed)
GLM full	36.9	317412	0.032	34.9	694	0.017
GLM reduced	36.3	323471	0.013	35.4	964	0.001
ESF full	39.1	337540	0.368	31.4	569	0.330
ESF reduced	40.4	324220	0.453	33.7	785	0.028
Wang’s model	35.9	291739	< 0.001	53.3	1312	< 0.001

*W is the sum of ranks assigned to differences with positive signs.

## Discussion

Our predictions of TSR across eastern USA obtained high deviance explained, non-clustered residuals, and comparable geographic patterns to observed TSR. This was accomplished by using gridded FIA point-sample-based TSR as the outcome variable, FA and MFDF as predictor variables, and ESF as the regression model. We discuss use of FIA data for TSR, performance of different models, spatial patterns of predicted TSR, and meanings of the chosen variables.

FIA point-sample data provided a reliable geographic record of TSR as it came from spatially explicit plot locations. In contrast, species range maps used in eight studies of TSR discussed in the Introduction have unknown amounts of spatial generalization that result in increased spatial autocorrelation that artificially inflates variance explained by environmental models [18]. That said, similar spatial patterns of TSR occur in maps created using FIA data (Fig 2C) and using Little’s range maps (Fig 1B, [10]; Fig 3A, [12]): lower TSR in northern than southern states, and regionally lower TSR in the Midwest prairie peninsula, along the lower Mississippi River and Gulf coast, and especially in Florida. One key difference is less spatial clustering of TSR from FIA point-sample data than from species range maps. Lower spatial clustering of TSR from FIA data likely reflects influence of local factors [7,39], something not reflected in spatially generalized range maps.

The geographic features just described in maps of TSR from gridded FIA data and Little’s maps were all reproduced in maps of TSR predicted by our ESF models. GLM models, however, did not predict the lowered TSR in the Midwest prairie peninsula nor along the Gulf coast. The GLM predictions were still an improvement on those of Wang et al. [12] that, in addition to not predicting lowered TSR in the prairie peninsula nor along the lower Mississippi River, also incorrectly predicted high TSR in Florida and along the Gulf coast. Of the seven other studies that developed environmental models of TSR, only three others presented maps of their TSR input data [6,9,10] and none mapped their TSR predictions. Our mapped output highlights the value of ESF models to help reproduce geographic realism in predicted TSR.

The full ESF model’s deviance explained of 87.7% was strong, exceeded by only two of the seven multivariate models of TSR described in the Introduction [8][13]. However, the deviance explained in TSR by our full GLM was lower than that by GLMs in seven other studies. Two likely reasons for weaker performance of our full GLM are multicollinearity and scale. First, our use of LASSO selected predictors with minimal multicollinearity, while stepwise regression used in other TSR models often chooses multicollinear predictors that artificially increase variance explained [40]. Second, our study area is smaller than the seven other TSR studies and, since smaller areas have a smaller range of TSRs and predictor variables, they have lower variance explained [7,39].

The importance of PSN in this study, the most influential predictor in all four multivariate models, and third in influence in individual GLMs, was unexpected as it was the fifth weakest of the 25 predictors used by Wang et al. [12] the only other TSR study that employed it. The negative relation between TSR and PSN was also unexpected given positive relations between PSN, net primary productivity and TSR: Robinson et al. [41] found net primary productivity to be positively related to PSN, and Adams & Woodward [8] found NPP to be positively related to TSR, and thus PSN and TSR should also be positively correlated. Supporting a positive relationship is the suggestion that an increase in PSN should increase niche availability [42] and thus species richness. In contrast, Cavanaugh et al. [43] found no relation between PSN and genus diversity of trees in tropical areas. It would be valuable to resolve this contradiction as changes in precipitation rather than temperature are the strongest driver of the changes in tree species distributions in the eastern USA [44].

MPDQ was the second strongest predictor in individual GLMs, in ESF full and GLM reduced models, and 3^rd^ strongest in ESF reduced and GLM full models. In contrast, it was 15^th^ strongest of 25 predictors in Wang et al. [12]. MPDQ suggests the importance of reducing physiological stress; the spatial pattern of MPDQ is the inverse of that for PSN, such that areas with high MPDQ having low PSN (S1 Fig). MPDQ has also been found to have a strong positive relation with oak species diversity [45].

MFDF was the strongest predictor in individual GLMs, second strongest in the full GLM, and fourth strongest predictor in the full ESF model. The negative relation between MFDF and TSR concurs with the killing effect frost has on tropical tree species and on the reduced growth rates that temperate trees incur to increase frost resistance [3]. The stronger relation between TSR and MFDF than with MTCQ, the strongest predictor of TSR for Wang et al. [12], was likely due to a non-linear relation between MTCQ and MFDF (S2 Fig). While MTCQ and MFDF were linearly related for all of Eastern USA, the slope of the relation was weaker in Florida with increasing MTCQ causing little reduction in MFDF. Thus, while MFDF showed a latitudinal pattern similar to that for MTCQ, the maritime influence on peninsular Florida was more meaningfully expressed in MFDF. Inclusion of MFDF in the full ESF model was thus critical in it being the only model to successfully predict Florida TSR. Note: MFDF was also selected by LASSO as the strongest predictor for just those grids with ≥41 and ≥51 FIA plots (S1 Table).

FA was the fourth strongest predictor in individual GLMs, third strongest in the full ESF, and fourth strongest in the full GLM. Low FA occurs in the areas with low TSR predicted by ESF and low TSR observed in Little’s maps (e.g. Montoya et al. [10]): the Midwest prairie peninsula, the lower Mississippi River, the Gulf Coast, and Florida (S1 Fig). Low TSR in those areas has been related to climatic factors similar to those in our models, but also edaphic and hydrographic factors not included in ours [46]. Low TSR there might also be due to a decrease in FA reducing the number of habitats and thus the number of species that can survive [21]. That these regional low-TSR features could be predicted by the ESF models and not by GLMs points out the value of accounting for spatial autocorrelation. The relations between FA and TSR are unlikely to be due to grids with more FA also having more FIA plots for two reasons. First, our bootstrapped estimates of TSR resampled the same number of plots (31) in all grids. Second, variable selection with LASSO for just grids with ≥41 plots and ≥51 plots also chose FA as its fourth strongest predictor (S2 Table).

The ESF models in two manners excelled in predicting the spatial pattern of TSR. First, they predicted regional variations in TSR such as the Midwest prairie peninsula and the Gulf coast that were not predicted by GLMs that employed the same variables. Second, standardized residuals were strongly clustered for GLMs, and slightly dispersed for ESF models. Clustering in GLMs highlights that the spatial autocorrelation present in outcome and predictor variables, when combined with spatially autocorrelated outcome variables from species range maps, can result in high variance explained but poor spatial predictions [18]. In contrast, slight dispersion in ESFs standardized residuals may reflect a tendency for ESF to over-correct spatial autocorrelation in geo-referenced data when too many eigenvectors are selected and added to the model [37]. Analyses conducted using only grids with ≥41 and ≥51 plots provided identical results in terms of the greater predictive power of ESF models, predictors chosen by LASSO, and the ability of ESF to best-predict TSR in Florida and Eastern inland USA (S3 Table).

## Supporting information

S1 Fig Maps of the predictor variables chosen by LASSO: FA, MFDF, MPDQ, PSN. (PDF)

S2 Fig Relations between MTCQ and MFDF for the 1234 grids with ≥31 plots. (PDF)

S1 Table GLM-based relations between the 18 individual predictor variables and tree species richness for grids with >41 and >51 plots. (PDF)

S2 Table Variables chosen by the LASSO method for grids with >41 and >51 plots. (PDF)

S3 Table Statistical analyses for grids with ≥41 and ≥51 plots per grid. (PDF)

## Abbreviations

ESF: Eigenvector Spatial Filtering
FA: Forest Area
MFDF: Mean Frost Day Frequency
TSR: Tree Species Richness
