# Supplementary figures and images for: Tree species richness predicted using a spatial environmental model including forest area and frost frequency, eastern USA
Source: PLoS One. 2018 Sep 18;13(9):e0203881. doi: 10.1371/journal.pone.0203881 (PMC6143234; doi:10.1371/journal.pone.0203881)

Appendix S1 Figure.

S1 Fig. Maps of the predictor variables chosen by LASSO: PSN, MPDQ, MFDF, FA.

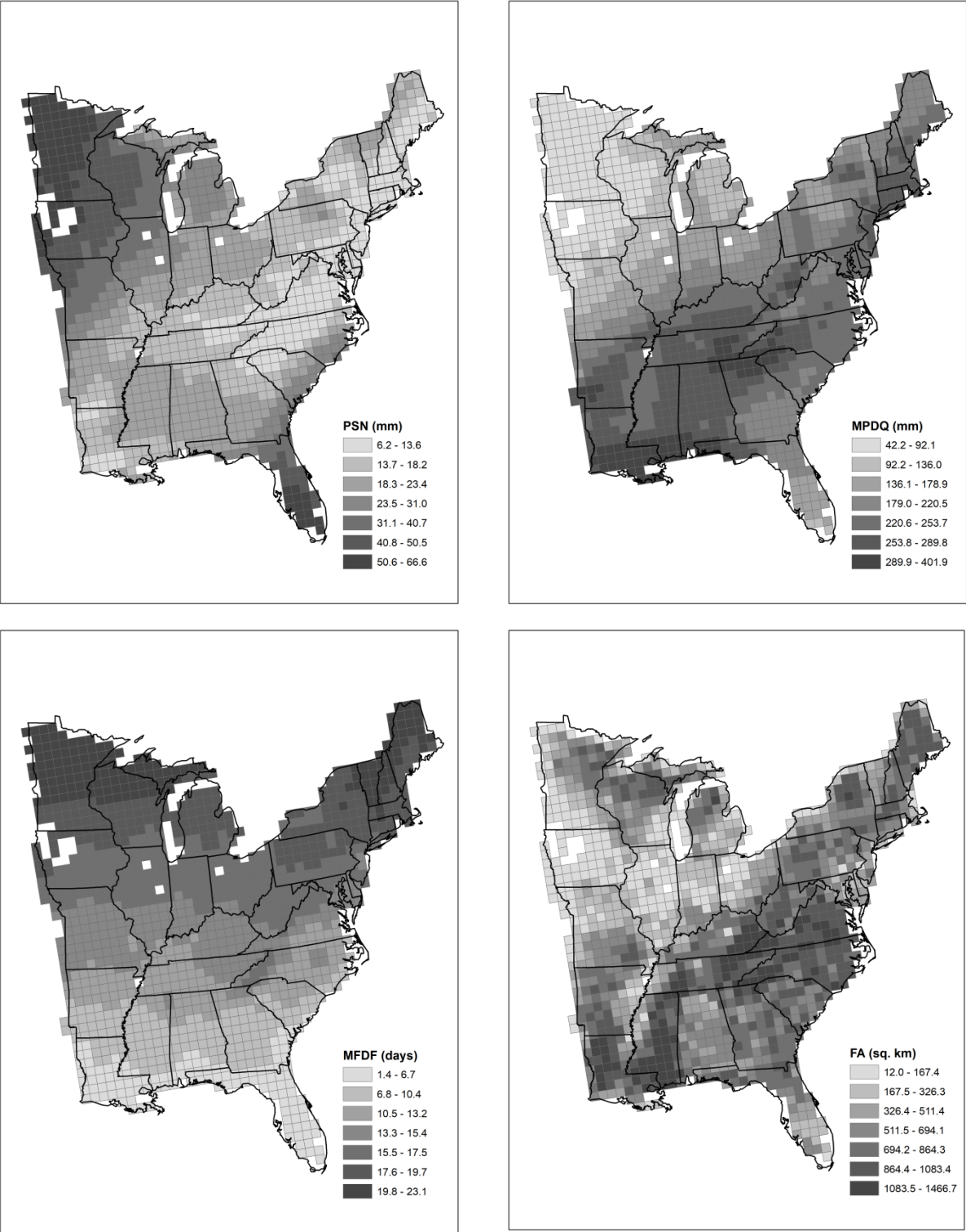

Supplement: S1 Fig — (PDF) [file pone.0203881.s001.pdf]
