# Supplementary material for: Tree species richness predicted using a spatial environmental model including forest area and frost frequency, eastern USA
Source: PLoS One. 2018 Sep 18;13(9):e0203881. doi: 10.1371/journal.pone.0203881 (PMC6143234; doi:10.1371/journal.pone.0203881)
Supplement: S2 Fig — (PDF) [file pone.0203881.s002.pdf]

APPENDIX S2 Figure.

S2 Fig. Relations between MTCQ and MFDF for the 1234 grids with ≥31 plots.

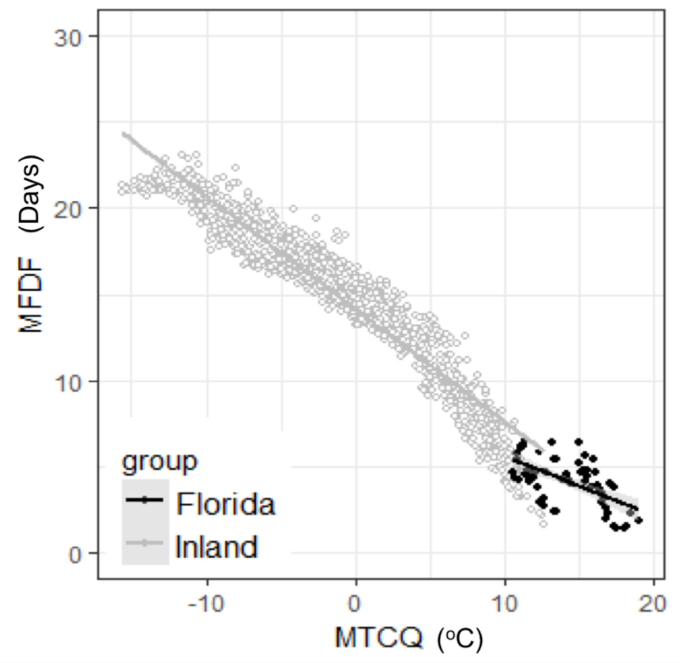

Linear regression equations: y = -0.344 x + 9.05 (Florida); y = -0.652 x + 14.16 (Inland without Florida). The slopes are significantly different (F:12047, P<0.001).
