# Supplementary material for: Tree species richness predicted using a spatial environmental model including forest area and frost frequency, eastern USA
Source: PLoS One. 2018 Sep 18;13(9):e0203881. doi: 10.1371/journal.pone.0203881 (PMC6143234; doi:10.1371/journal.pone.0203881)
Supplement: S1 Table — (PDF) [file pone.0203881.s003.pdf]

APPENDIX S1 Table.

S1 Table. GLM-based relations between the 18 individual predictor variables and tree species richness for grids with ≥41 and ≥51 plots.

Category	Variable	Plot Count ≥41		Plot Count ≥51	
		Standardized coefficient	Deviance (%) explained	Standardized coefficient	Deviance (%) explained
Areal factors	FA	0.14	27.87	0.14	27.60
	WA	-0.10	12.33	-0.09	12.29
Climatic seasonality	ART	-0.13	21.11	-0.13	20.20
	PSN	-0.19	33.81	-0.17	31.53
	TSN	-0.12	21.72	-0.11	21.75
Energy availability	MAT	0.13	24.51	0.12	23.99
	MTWQ	0.12	24.81	0.13	24.99
	PET	0.13	25.45	0.14	26.76
Energy-water dynamic	PET – PET2 + MAP	0.09	17.72	0.10	19.72
Habitat heterogeneity	RA	0.02	0.62	0.02	0.63
	RMAP	-0.11	19.12	-0.12	20.07
	RMAT	-0.13	23.29	-0.12	20.81
Limiting climatic factors	MFDF	-0.16	32.12	-0.16	29.12
	MPDQ	0.18	35.21	0.17	33.21
	MTCQ	0.12	22.81	0.12	25.13
Water availability	AET	0.12	26.13	0.14	26.81
	AI	0.00	0.02	0.00	0.01
	MAP	0.13	23.36	0.11	21.77
