# Supplementary material for: Tree species richness predicted using a spatial environmental model including forest area and frost frequency, eastern USA
Source: PLoS One. 2018 Sep 18;13(9):e0203881. doi: 10.1371/journal.pone.0203881 (PMC6143234; doi:10.1371/journal.pone.0203881)
Supplement: S2 Table — (PDF) [file pone.0203881.s004.pdf]

APPENDIX S2 Table.

S2 Table. Variables chosen by the LASSO method for grids with ≥41 and ≥51 plots.

Full model (variables selected from all 18 predictors)			Reduced model (variables selected from the 16 predictors other than MFDF and FA)		
Selected variables	Plot ≥41	Plot ≥51	Selected variables	Plot ≥41	Plot ≥51
	Standardized penalized coefficient			Standardized penalized coefficient	
MFDF	-0.0066415	-0.0054315	PSN	-0.0054455	-0.0056451
PSN	-0.0054529	-0.0050063	PET	0.0017162	0.0011144
MPDQ	0.0004175	0.0004168	MPDQ	0.0002735	0.0001963
FA	0.0001251	0.0001649	MTCQ	0.0001537	0.0001953

Variables are ordered from highest to lowest absolute standardized coefficient.
