# Supplementary material for: Tree species richness predicted using a spatial environmental model including forest area and frost frequency, eastern USA
Source: PLoS One. 2018 Sep 18;13(9):e0203881. doi: 10.1371/journal.pone.0203881 (PMC6143234; doi:10.1371/journal.pone.0203881)
Supplement: S3 Table — (PDF) [file pone.0203881.s005.pdf]

APPENDIX S3 Table.

S3A Table. GLM Model comparisons between grids with ≥41 and ≥51 plots.

Full models (variables selected from all 18 predictors)						
Variables	GLM Poisson (Plot ≥41)			GLM Poisson (Plot ≥51)		
	Standardized Coefficient	Standard Error	VIF	Standardized Coefficient	Standard Error	VIF
(Intercept)	3.356441	0.003545***	NA	3.756345	0.002435***	NA
PSN	-0.17542	0.006377***	3.66	-0.19942	0.005427***	3.66
MFDF	-0.14111	0.003834***	2.75	-0.12232	0.003624***	3.85
MPDQ	0.11345	0.014351 ***	4.15	0.12025	0.012311 ***	4.18
FA	0.07535	0.004676***	4.67	0.07536	0.00654***	4.23
AIC	9536			9112		
Null Deviance	5664			3865		
Residual Deviance (% explained)	2234 (60.55%)			1344 (65.22%)		
Observed Moran's I	0.76			0.79		
Z-score of Moran's I	21.332***			21.435***		
# selected eigenvectors	-			-		
Reduced models (variables selected from the 16 predictors other than MFDF and FA)						
Variables	GLM Poisson (Plot ≥41)			GLM Poisson (Plot ≥51)		
	Standardized Coefficient	Standard Error	VIF	Standardized Coefficient	Standard Error	VIF
(Intercept)	3.1336	0.00451***	NA	3.1336	0.00643***	NA
PSN	-0.1852	0.012339***	3.61	-0.1788	0.01349***	2.61
MPDQ	0.13753	0.01726***	3.34	0.15453	0.01345***	2.64
PET	0.10428	0.003432**	2.42	0.17543	0.00232**	2.82
MTCQ	0.08433	0.013236***	4.63	0.08292	0.01996***	2.63
AIC	9959			10221		
Null Deviance	5664			3865		
Residual Deviance (% explained)	2865 (49.41%)			2122 (45.09%)		
Observed Moran's I	0.74			0.72		
Z-score of Moran's I	25.37***			25.37***		
# selected eigenvectors	-			-		

Variables are ordered by in descending order of the absolute size of the standardized coefficient. VIF is the variance inflation factor. Significance level: ***0.001 **0.01 *0.05

S3B Table. ESF Model comparisons between grids with ≥41 and ≥51 plots.

Full models (variables selected from all 18 predictors)						
Variables	ESF (Plot ≥41)			ESF (Plot ≥51)		
	Standardized Coefficient	Standard Error	VIF	Standardized Coefficient	Standard Error	VIF
(Intercept)	2.96252	0.0642652***	NA	2.34552	0.0642652***	NA
PSN	-0.26425	0.0036234***	2.30	-0.29425	0.0042334***	2.77
MPDQ	0.16972	0.0193452***	3.68	0.13273	0.0145682***	3.28
FA	0.14532	0.0152443**	3.75	0.13530	0.0163453**	3.21
MFDF	-0.12642	0.0047546**	1.31	-0.1342	0.003426***	1.33
AIC	7623			7867		
Null Deviance	5664			3865		
Residual Deviance (% explained)	654 (88.45%)			544 (85.92%)		
Observed Moran's I	0.17			0.17		
Z-score of Moran's I	2.243*			2.215*		
# selected eigenvectors	166			154		
**Reduced models (variables selected from the 16 predictors other than MFDF and FA)**						
Variables	ESF (Plot ≥41)			ESF (Plot ≥51)		
	Standardized Coefficient	Standard Error	VIF	Standardized Coefficient	Standard Error	VIF
(Intercept)	3.0445	0.01765***	NA	3.1424	0.01335***	NA
PSN	-0.22955	0.017235***	4.54	-0.23436	0.016455**	2.54
PET	0.08246	0.003867**	1.57	0.04533	0.005467**	1.52
MPDQ	0.07753	0.006346**	2.24	0.07642	0.006346**	2.21
MTCQ	0.04654	0.004456***	1.44	0.03464	0.007545***	1.28
AIC	8003			8021		
Null Deviance	5664			3865		
Residual Deviance (% explained)	989 (82.53%)			687 (82.25%)		
Observed Moran's I	-0.12			-0.11		
Z-score of Moran's I	2.563**			2.249**		
# selected eigenvectors	145			165		

Variables are ordered by in descending order of the absolute size of the standardized coefficient. VIF is the variance inflation factor. Significance level: ***0.001 **0.01 *0.05

S3C Table. Wilcoxon signed rank test between FIA and predicted TSR for eastern US and Florida for grids with ≥41 and ≥51 plots.

Model (Plot ≥41)	Eastern US inland (Median= 42.2, N = 917)			Florida (Median = 34.4, N = 28)		
	Median	Wilcoxon signed rank test (W*)	P- value (two-tailed)	Median	Wilcoxon signed rank test (W*)	P- value (two-tailed)
GLM full	38.3	342342	0.013	35.2	624	0.011
GLM reduced	39.5	333471	0.011	36.5	654	0.001
ESF full	41.2	345431	0.352	34.1	459	0.313
ESF reduced	42.5	343120	0.556	35.7	675	0.012
(Plot ≥51)	Eastern US inland (Median= 44.3, N = 575)			Florida (Median = 36.1, N = 21)		
GLM full	38.0	353160	0.023	34.5	454	0.024
GLM reduced	38.8	327720	0.011	36.6	874	0.000
ESF full	42.9	387548	0.422	34.7	669	0.255
ESF reduced	44.8	326688	0.431	35.2	545	0.021

*W value is the sum of ranks assigned to the differences with positive sign.
